# Alterations in DNA methylation machinery in a rat model of osteoarthritis of the hip

**DOI:** 10.1186/s13018-024-04847-0

**Published:** 2024-06-16

**Authors:** Yuya Kawarai, Junichi Nakamura, Shigeo Hagiwara, Miyako Suzuki-Narita, Kazuhide Inage, Seiji Ohtori

**Affiliations:** 1 Department of Orthopaedic Surgery, Graduate School of Medicine, Chiba University, 1- 8-1 Inohana, Chuo-ku, Chiba City, Chiba 260-8677 Japan; 2 Department of Bioenvironmental Medicine, Graduate School of Medicine, Chiba University, 1- 8-1 Inohana, Chuo-ku, Chiba City, Chiba 260-8677 Japan

**Keywords:** Hip osteoarthritis, Rat MIA-induced OA model, Epigenetics, DNA methylation

## Abstract

**Background:**

This study aimed to validate alterations in the gene expression of DNA methylation-related enzymes and global methylation in the peripheral blood mononuclear cell (PBMC) and synovial tissues of animal hip osteoarthritis (OA) models.

**Methods:**

Animals were assigned to the control (no treatment), sham (25 µL of sterile saline), and OA (25 µL of sterile saline and 2 mg of monoiodoacetate) groups. Microcomputed tomography scan, histopathological assessment and pain threshold measurement were performed after induction. The mRNA expression of the DNA methylation machinery genes and global DNA methylation in the PBMC and hip synovial tissue were evaluated.

**Results:**

The OA group presented with hip joint OA histopathologically and radiologically and decreased pain threshold. The mRNA expression of DNA methyltransferase (*Dnmt 3a*), ten–eleven translocation (*Tet*) *1* and *Tet 3* in the synovial tissue of the OA group was significantly upregulated. Global DNA methylation in the synovial tissue of the OA group was significantly higher than that of the control and sham groups.

**Conclusions:**

The intra-articular administration of monoiodoacetate induced hip joint OA and decreased pain threshold. The DNA methylation machinery in the synovial tissues of hip OA was altered.

## Introduction

Osteoarthritis (OA) is among the most prevalent musculoskeletal diseases. In 2020, it affected approximately 595 million people worldwide, which is equivalent to approximately 8% of the global population. The incidence rates of osteoarthritis are projected to increase to 75% in the knee and 79% in the hip by 2050 [1]. To date, there are no disease-modifying agents that can prevent or control OA development, resulting in an increase in the number of patients with OA eventually requiring joint replacement surgery. Therefore, the OA pathophysiology should be completely elucidated, and novel treatments must be developed. OA is a multifactorial disease, and its associated risk factors include aging [2], sex [3], race [4], obesity [5], dietary changes [5, 6], trauma history [7], occupation [8], and genetics [9]. In recent years, there has been a growing interest in epigenetics, which links genetics, a factor in the development of congenital OA, and lifestyle, an acquired factor [10].

Based on epigenetics, several mechanisms can modify gene expression without causing changes to the underlying DNA sequence [11]. DNA methylation at the C5 position of cytosine in CpG dinucleotides, which is the most commonly evaluated factor, is among the representative epigenetic mechanisms [12]. Several studies have reported that this modification is not static. Rather, it is dynamically affected by environmental factors, such as aging, diet, and exercise [13, 14]. DNA methylation mainly involves adding a methyl group to the 5′ carbon of the cytosine pyrimidine ring of CpG dinucleotides, which act as a repressive signal and inhibit gene transcription activity. DNA methyltransferases are enzymes that transfer methyl groups to the DNA. DNMT 1, DNMT 2, DNMT 3a, DNMT 3b, and DNMT 3 L are classified as members of the DNMT family. The primary function of DNMT 2 is to catalyze the methylation of RNA. Meanwhile, DNMT 3 L does not have a catalytic activity. Thus, CpG methylation is determined by the following DNA methyltransferases: DNMT1, DNMT 3a, and DNMT 3b. DNMT 1 is responsible for maintaining methylation during DNA replication and damage repair, and DNMT 3a and DNMT 3b play a major role in *de novo* methylation [15]. Enzymes of the ten–eleven translocation (TET) family, such as TET 1, TET 2, and TET 3, catalyze the stepwise oxidation of 5-methylcytosine (5-mC) in the DNA to 5-hydroxy-methylcytosine and further promote product oxidation. Moreover, they are responsible for methylation homeostasis [16]. Previous animal studies have reported that trauma and dietary changes alter DNA methylation and induce OA. The association between epigenetic changes and OA has attracted attention [17, 18]. Moreover, a recent matched case-control study identified a connection between DNMT polymorphisms and primary knee OA [19].

Genetic variation related to OA pathology commonly occurs in the noncoding regions of the genome [20]. Thus, most genetic variations affect gene regulation that alters protein expression rather than the function of the protein by changing its original sequence [21]. Thus, increasing studies have focused on epigenetics, particularly DNA methylation, which regulates gene expression without changing the gene sequence [22]. There are a growing number of reports on the use of epigenetics for assessing DNA methylation as a representative mechanism. Several studies have focused on the DNA methylation of individual genes in the cartilage, one of the main OA pathologies [23, 24]. However, all CpG sites within a gene’s regulatory region cannot be analyzed. Moreover, previous studies have investigated genes already associated with OA. Therefore, it is challenging to identify novel pathways that can be essential for a deeper understanding of OA pathogenesis. To understand the effects of methylation in OA, it is important to examine not only the expression of individual genes but also the expression of enzymes that regulate DNA methylation, such as DNMTs and TETs. Nevertheless, the pathological changes in OA encompass progressive degeneration of the articular cartilage, thickening of the subchondral bone, osteophyte formation, and inflammation of the synovial tissue. However, previous studies have commonly investigated the gene expression of enzymes that regulate DNA methylation in the cartilage. To date, only a few studies have focused on the use of synovial tissue, which is used to assess the underlying OA pathologies, and blood, which can be easily evaluated in clinical settings.

Previous studies have developed hip OA animal models [25–27]. This study aimed to validate alterations in the gene expression of DNA methylation-related enzymes and global methylation in the synovial tissue and peripheral blood mononuclear cell (PBMC) tissue in hip OA animal models.

## Materials and methods

### Animals

All research protocols were approved by our institutional ethics committee (#4-118) and were in accordance with the National Institutes of Health Guidelines for the Care and Use of Laboratory Animals.

Male Sprague Dawley rats weighing 200–300 g were used (CLEA, Tokyo, Japan) in this experiment. The number of animals required for this experiment was determined using the resource equation method [28]. The animals were housed in a semibarrier system with a controlled environment (12-h/12-h light/dark cycle, temperature: 21–23 °C, and humidity: 45–65%). The rats had unrestricted access to food and water upon arrival at the facility. All rats were fed with a standard rodent diet (CRF-1; Oriental Yeast Co., Ltd., Tokyo, Japan).

### Intra-articular injection of monoiodoacetate into the hip joint

All rats were anesthetized via the intraperitoneal injection of 0.3 mg/kg of medetomidine (Nippon Zenyaku Kogyo Co., Ltd., Tokyo, Japan), 4.0 mg/kg of midazolam (Maruishi Pharmaceutical Co., Ltd., Osaka, Japan), and 5.0 mg/kg of butorphanol (Meiji Seika Pharma Co., Ltd., Tokyo, Japan), according to previously reported methods [29]. The analgesic agents medetomidine, which acts as an analgesic by stimulating alpha-2 receptors, and butorphanol injection were selected. During surgery, the animals were carefully monitored to ensure that they were not in pain. All procedures were performed aseptically. The following solutions were injected into the right hip joint of each rat using the posterior approach [25, 30]: 25 µL of sterile saline and 2 mg of monoiodoacetate (MIA) (Sigma-Aldrich, St. Louis, MO, the USA) (OA group, *n* = 18) and 25 µL of sterile saline (sham group, *n* = 18). In total, 18 rats were untreated (control group). The injected agent was restricted to the joint cavity, as described in a previous study [25]. The MIA dose was determined according to a previous report [26].

### Microcomputed tomography scan

The rats were positioned in a chamber. A mixture of oxygen and 1.5% sevoflurane vapor (Mylan Inc., Canonsburg, PA, the USA) was delivered at a flow rate of 3.0 L/min. The sevoflurane concentration was gradually increased to obtain the target anesthetic depth [31]. CT scan was performed before and 2 and 4 weeks after MIA administration. The right hip joints were scanned using a CosmoScan FX (Rigaku Corp., Tokyo, Japan) at 360° rotation. The X-ray source was set at an energy of 90 kV and intensity of 88 mA with a field of view of 45 mm and an imaging time of 18 s. The imaging area was the whole hip joint. Coronal and axial images, including the center of the femoral head, were used for evaluation. The radiographic assessment results were classified using the Kellgren and Lawrence (KL) classification system [32, 33] (Table 1).

**Table 1:** Kellgren-lawrence grading system for osteoarthritis

Grade	Radiological findings
0	No radiological findings of osteoarthritis
I	Doubtful joint space narrowing and possible osteophyte
II	Presence of definite joint space narrowing, definite osteophytes, and slight sclerosis
III	Marked joint space narrowing, slight osteophytes, some sclerosis and cyst formation, and deformity of the femoral head and acetabulum
IV	Gross loss of joint space with sclerosis and cysts, marked deformity of the femoral head and acetabulum, and large osteophytes

### Histopathological findings

Samples were collected 28 days after the administration of MIA or saline. The animals were intraperitoneally anesthetized and perfused transcardially as previously described [27]. The tissues around the right hip joint, including the bone, cartilage, synovium, and capsule, were harvested. The resected limbs were resected at the mid-femur and the center of the femoral head immersed in 10% neutral buffered formalin for 3 days. The specimens were continuously demineralized in K-CX reagent (FALMA, Tokyo, Japan) for 30 h and 5% sodium sulfate for 16 h. Then, they were paraffin embedded for subsequent coronal sectioning. The samples were serially sectioned in steps of 8 μm and stained using hematoxylin and eosin, safranin O, and toluidine blue. Osteoarthritic changes were evaluated using the Osteoarthritis Research Society International (OARSI) histopathological score [34]. For each joint, 10 slices centered on the maximum diameter of the femoral head were scored. Each sample was assessed using the depth (grading) and width (staging) of the osteoarthritic changes. The score was finally expressed by multiplying the depth and width. The average scores of each group were compared.

### Pain threshold measurement

Pain threshold assessment was conducted to evaluate pain, which is the primary symptom of OA, to confirm the validity of this OA model and rule out other conditions such as Charcot arthropathy. Using the von Frey assay, mechanical plantar skin sensitivity was evaluated. The animals were habituated to the testing room for 60 min and moved to the testing apparatus. They were also kept on the apparatus for another 60 min before the measurements. All tests were conducted between 9:00 am and 3:00 pm. The experimenter was blinded to each group. The von Frey tests have been extensively described in a previous study [35]. Mechanical sensitivity was determined using the up–down technique with von Frey filaments (Mono-filament kit; Smith & Nephew, Germantown, WI) on the right hind paw plantar surface [36]. The testing was performed before (baseline) and 7, 14, and 28 days after induction. The von Frey filaments were applied for 4 s or until paw withdrawal. Results were expressed as 50% threshold to withdraw in grams, as described in a previous study [35]. The stimulus intensity ranged from 1 g to 60 g, corresponding to filament numbers (4.08, 4.17, 4.31, 4.56, 4.74, 4.93, 5.07, 5.18, 5.46, and 5.88).

### RNA extraction and quantitative real-time polymerase chain reaction

Total RNA was extracted from the PBMCs and synovial tissues with the acid guanidinium thiocyanate–phenol–chloroform method using the ISOGEN RNA extraction kit (Nippon Gene, Tokyo, Japan) 28 days after induction. Peripheral blood sample was collected from each rat, and PBMCs were isolated via Ficoll-Paque™ PLUS gradient centrifugation (GE Healthcare, Chicago, IL, the USA). The PBMCs were homogenized while immersed in 0.75 mL of ISOGEN-LS. In total, 25 mg of synovial tissue was collected under direct visualization from the hip joint of the affected side of each rat. Synovial samples were immediately crushed in liquid nitrogen using a mortar and pestle and were immersed in 1 mL of ISOGEN. They were completely homogenized using a pellet pestle motor (Sigma-Aldrich, St. Louis, MO, USA; Z359971) on ice. Chloroform was added to each tube, and samples were centrifuged for 15 min at 12,000 × g at 4 °C. The RNA-containing aqueous phase was obtained via centrifugation. RNA was precipitated via centrifugation in 0.8 mL of isopropanol, purified with 70% ethanol, and dissolved in deionized distilled water to obtain the total RNA solution. The RNA quality was assessed using the NanoDrop 2000 spectrophotometer (Thermo Fisher Scientific, Waltham, MA, the USA) and RNA Pico Chip Bioanalyzer 2100 (Agilent, Santa Clara, CA, the USA). The RNA integrity number of all samples ranged from 7.0 to 9.2. In total, 500 ng of RNA from each sample was reverse-transcribed into single strands using the High Capacity cDNA Reverse Transcription kit (Applied Biosystems, Foster City, CA, the USA). A cDNA template was mixed with SsoAdvanced Universal SYBR Green Supermix (Bio-Rad, Hercules, CA, the USA), and quantitative polymerase chain reaction (qPCR) was performed using the CFX Connect Real-Time PCR System (Bio-Rad, Hercules, CA, the USA). Table 2 shows the primers used for qPCR. Each qPCR was conducted in duplicate for each sample. The mRNA expression was normalized to the average CT values of glyceraldehyde 3-phosphate dehydrogenase using the ΔΔCt method [37]. The fold-change relative expression was calculated with the control group as the reference group.

**Table 2:** Primer pairs used for quantitative PCR analysis

Gene name	Forward primer	Reverse primer
*Gapdh*	5’ CTCTCTGCTCCTCCCTGTTC3’	5’TACGGCCAAATCCGTTCACA3’
*Dnmt 1*	CAATGAGGCACTGTCCGTCT	AAGTGACCGCGACTGCAATA
*Dnmt 3a*	CTTCTCTGAAGCCCTCGCAG	CGCTCTTCCTTACCACGGTT
*Dnmt 3b*	ACAACCATTGACTTTGCCGC	CGTTCTCGGCTCTCCTCATC
*Tet 1*	CCAAAGATGGCTCTCCAGTT	GAGCTGAGTCAGTGCTTCTATG
*Tet 2*	CTGCCCTGTAGGATTTGTTAGA	GAGGGTAAGCTGCTGAATGT
*Tet 3*	TGGAGATTCAAGGCAGCTAAG	AAGTCGGGCTTCTGGTCTA

### Global DNA methylation analysis

Genomic DNA was extracted from the PBMC and synovial tissues 28 days after MIA administration. The PBMC and synovial tissues were harvested in the same manner as RNA extraction. The harvested synovial samples were immediately flash frozen in liquid nitrogen and stored at − 80 °C. They were homogenized in liquid nitrogen with a mortar and pestle for DNA extraction. Genomic DNA was extracted using the Qiagen DNeasy Blood & Tissue kit (Qiagen, Hilden, Germany), according to the manufacturer’s instructions. Global DNA methylation was measured using the MethylFlash Methylated DNA Quantification kit (Epigentek, Farmingdale, NY, the USA). In 100 ng of genomic DNA, methylated DNA was detected using capture and detection antibodies against 5-mC and then quantified colorimetrically by reading absorbance at 450 nm. Relative quantification was used to calculate the percentage of 5-mC (%5-mC) in the total DNA according to the manufacturer’s instructions. The fold-change relative expression was calculated with the control group as the reference group.

### Statistical analysis

All data were analyzed using GraphPad Prism version 9 (GraphPad Software, San Diego, CA, the USA). Data were analyzed using one-way analysis of variance, followed by Tukey’s *post hoc* test. A p value of < 0.05 was considered statistically significant. Data were analyzed as indicated in the figure legends.

## Results

### Microcomputed tomography scan and histological analysis

**Fig. 1:**
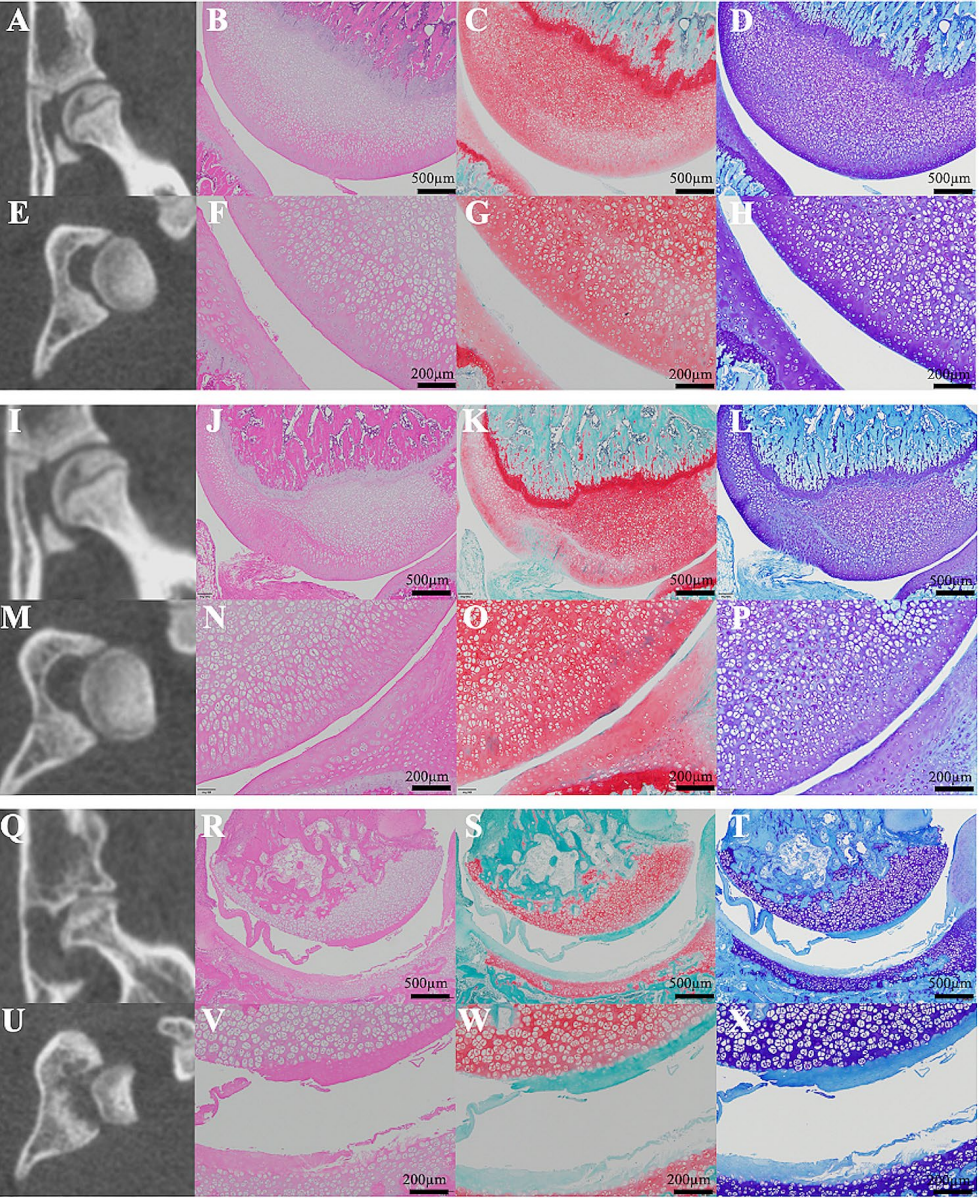
Representative microcomputed tomography (µ-CT) scan images and histopathological images of the hip on after induction day 28. The control group did not present with osteoarthritic changes in the coronal and axial µ-CT scan images (**A** and **E**). There was no evidence of degeneration or narrowing of the joint space on either low- or high-magnification images (**B**–**D** and **F**–**H**). The sham group did not present with evident osteoarthritic changes on the coronal and axial µ-CT scan images (**I** and **M**). A decrease in the number of superficial cells in the articular cartilage and minor superficial fibrillation with slight edema were observed (OARSI Grade 1, **J**–**L** and **N**–**P**). The OA group presented with significant joint space narrowing and subchondral bone cyst formation in the acetabulum, and the femoral head migrated proximally and laterally (**Q** and **U**). The surface tissue was detached over half of the articular surface, and it was free in the joint cavity, thereby indicating significant cartilage matrix loss (OARSI grade 4, R–T and V–X). From left to right are the µ-CT scan, hematoxylin and eosin, safranin O, and toluidine blue images

**Fig. 2:**
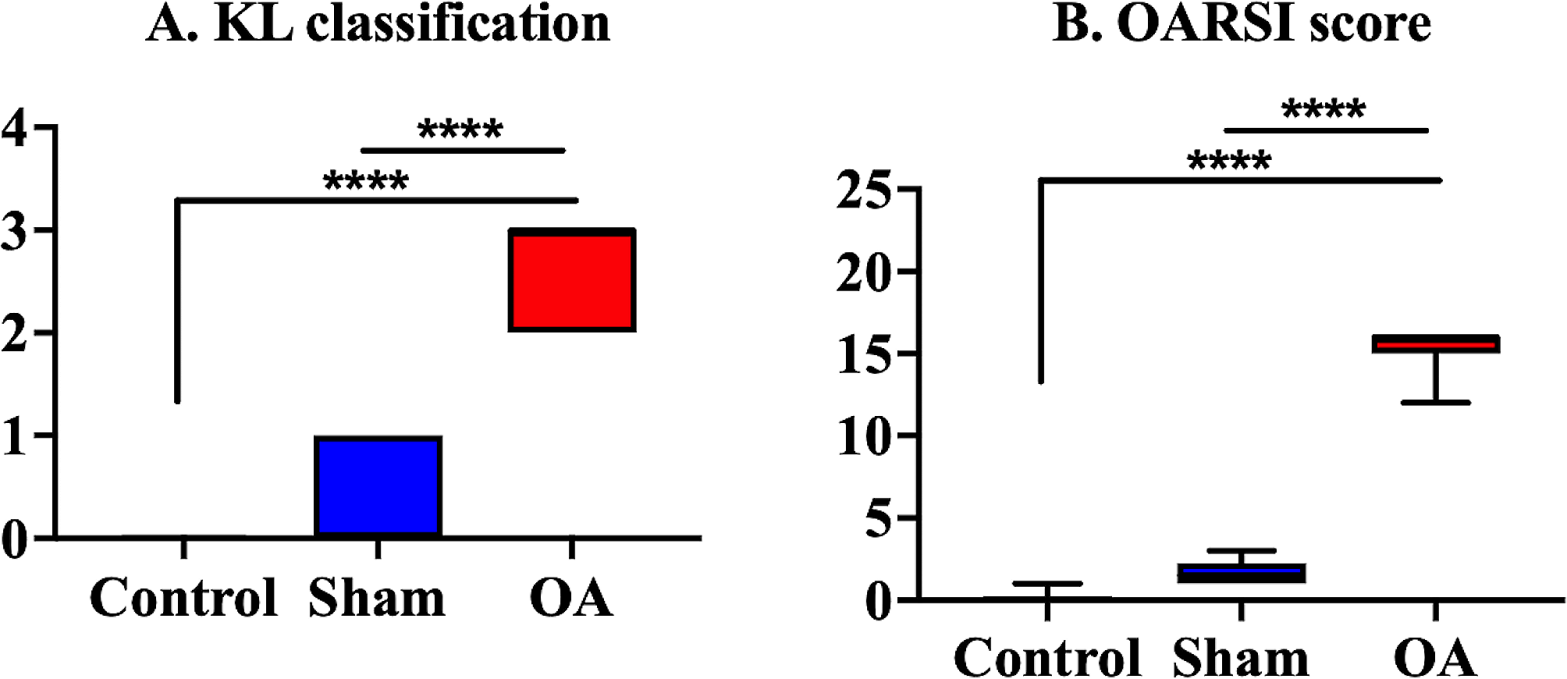
Radiographic and histopathological scoring. In the KL classification evaluation (**A**), the OA group had a significantly higher grade than the control and sham groups. In the histological analysis (**B**), the OA group exhibited a significant difference in the OARSI score compared with the other groups 28 days after induction. Unlike the control and sham groups, the OA group presented with significant osteoarthritic alteration. The medians are depicted using the horizontal lines inside the boxes. The 25th and 75th percentiles are shown as the bottoms and tops of the boxes, respectively. The minimum and maximum values are presented as the small horizontal lines below and above the boxes. *n* = 6 per group. Data were analyzed using the one-way analysis of variance, followed by the Tukey’s *post hoc* test. ****, *p* < 0.0001

The OA group presented with significant joint space narrowing (JSN) and subchondral bone cyst formation on µ-CT scan (Fig. 1Q and U). However, the control and sham groups did not present with osteoarthritic changes (Fig. 1A, E and I, and 1M). In the KL classification evaluation, the OA group had a significantly higher grade than the control and sham groups (Fig. 2A). Histological analysis revealed a decrease in the number of superficial cells in the articular cartilage and minor superficial fibrillation with slight edema in the sham group (OARSI grade 1, Fig. 1J–L and N–P). In the OA group, the surface tissue was detached over half of the articular surface, and it was free in the joint cavity, showing significant cartilage matrix loss. The deep surface was serrated (deep fibrillation) (OARSI grade 4, Fig. 1R–T and V–X). The OA group exhibited a significant difference in the OARSI score compared with those of the other groups 28 days after induction (control group: 0.17 ± 0.17, sham group: 2.11 ± 0.19, and OA group: 15.33 ± 0.67). The OA group presented with significant osteoarthritic alteration compared with the control and sham groups (Fig. 2B, *p* < 0.0001, control vs. OA group and sham vs. OA group, respectively).

### Pain threshold measurement

**Fig. 3:**
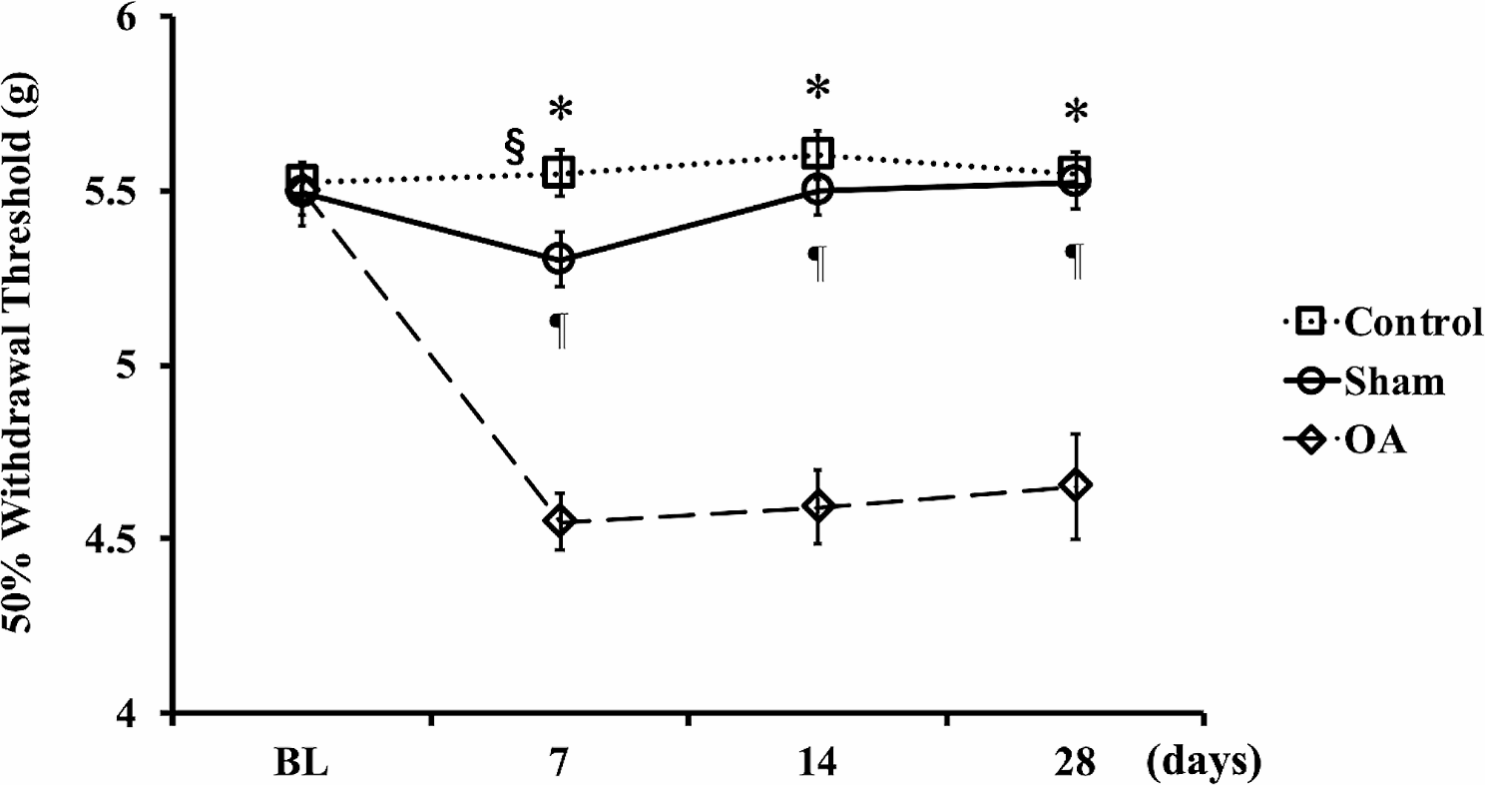
Alteration in hypersensitivity over time as determined using the von Frey assay in each group. The von Frey testing was performed before administration (baseline, BL). Then, the animals were injected with 2.0 mg of MIA (OA group) or saline (Sham group). However, the control group did not receive any treatment. They animals were tested 7, 14, and 28 days after induction. There was a significant difference between the control and sham groups on day 7 after MIA administration. In the OA group, mechanical hypersensitivity was observed from 7 to 28 days after administration. Data were presented as mean ± standard error of the mean, *n* = 18 per group. Mechanical sensitivity was analyzed using one-way analysis of variance, followed by the Tukey’s *post hoc* test. *, *p* < 0.05, control vs. OA group. ¶, *p* < 0.05, sham vs. OA group. §, *p* < 0.05, control vs. sham group

The OA group presented with a significantly lower pain threshold than the control and sham groups from 7 to 28 days after induction (Fig. 3). There was a significant difference between the control and sham groups on day 7 after MIA administration.

### mRNA expression of DNA methyltransferases and the ten–eleven translocation family in peripheral blood mononuclear cells

#### DNA methyltransferases

**Fig. 4:**
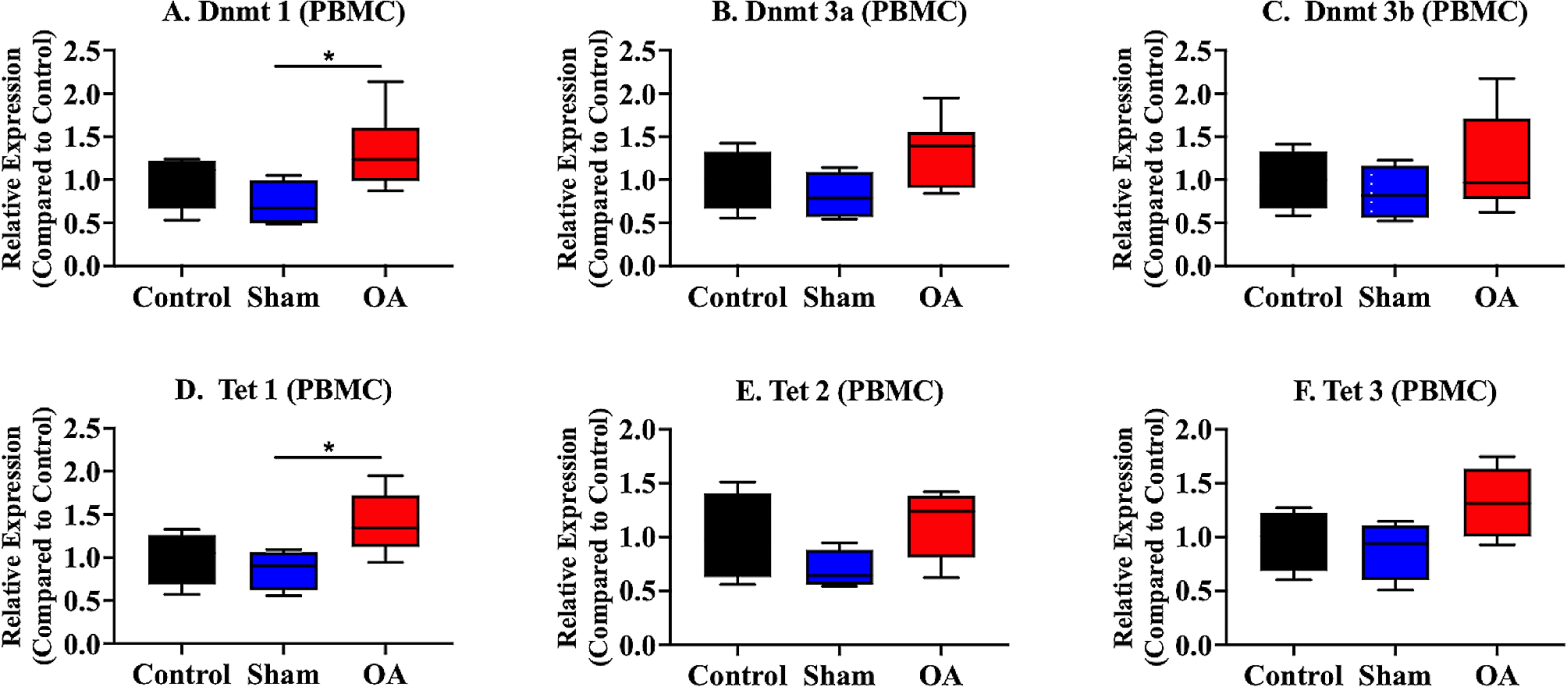
mRNA expression of DNA methyltransferases and the ten–eleven translocation family in the peripheral blood mononuclear cells. The OA group presented with significant increases in the mRNA expression of *Dnmt1* (**A**) and *Tet 1* (**D**) compared with that in the sham group. The medians are depicted using the horizontal lines inside the boxes. The 25th and 75th percentiles are shown as the bottoms and tops of the boxes, respectively. The minimum and maximum values are presented as the small horizontal lines below and above the boxes. *n* = 6 per group. Data were analyzed using one-way analysis of variance, followed by the Tukey’s *post hoc* test. *, *p* < 0.05. PBMC, peripheral blood mononuclear cell

The mRNA expression of the genes encoding *Dnmt1*, *Dnmt3a*, and *Dnmt3b* was measured. The mRNA expression of *Dnmt 1* in the OA group significantly increased compared with that in the sham group (Fig. 4A).

#### Ten–eleven translocation family

The mRNA expression of *Tet 1, Tet 2*, and *Tet 3* was evaluated. The mRNA expression of *Tet 1* in the OA group was significantly higher than that in the sham group (Fig. 4D).

#### mRNA expression of DNA methyltransferases and the ten–eleven translocation family in the synovial tissue from the hip joint

#### DNA methyltransferases

**Fig. 5:**
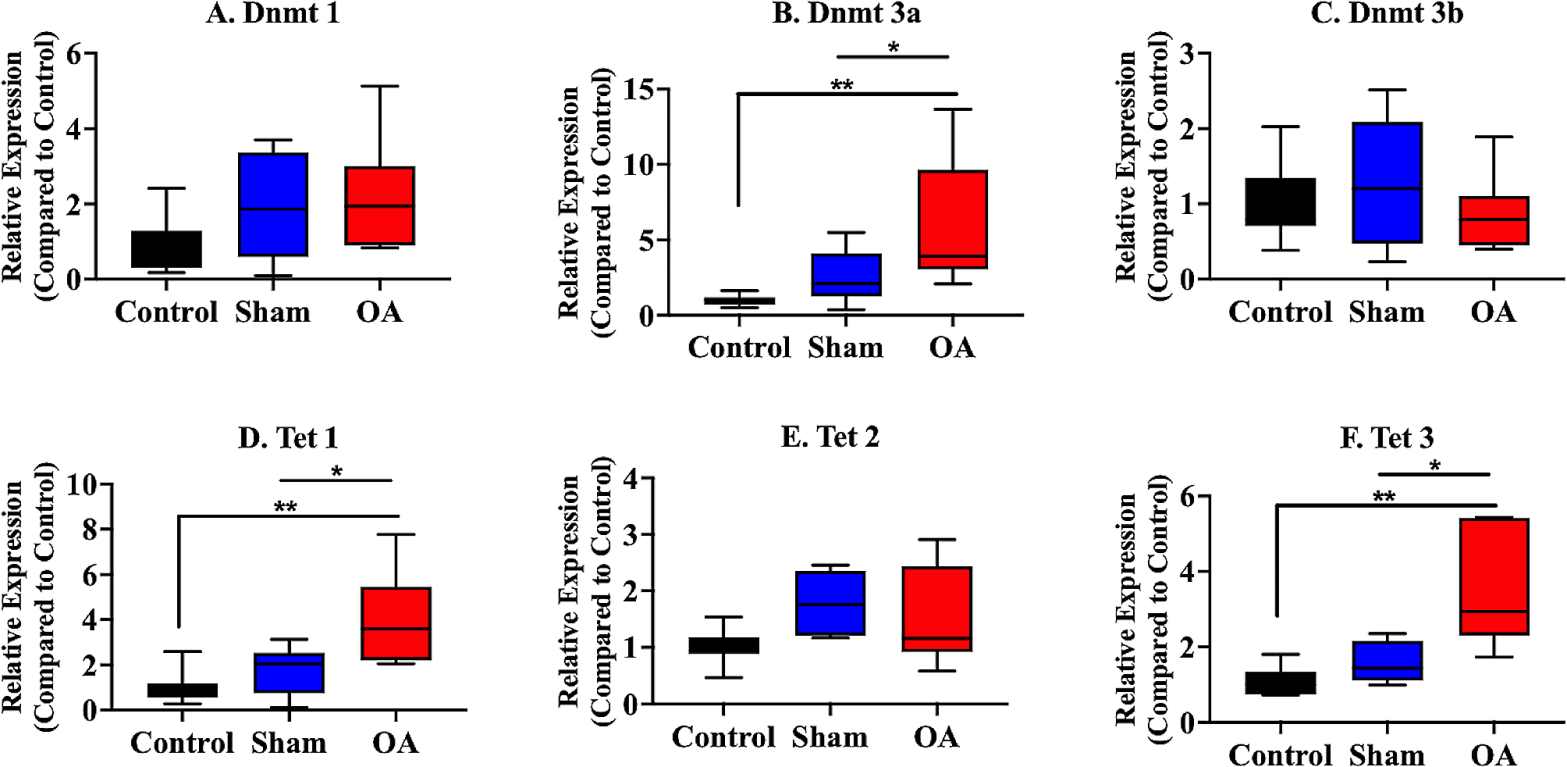
mRNA expression of DNA methyltransferases and the ten–eleven translocation family in the synovial tissue from the hip joint. The OA group presented with significant increases in the mRNA expression of *Dnmt3a* (B), *Tet 1* (**D**), and *Tet 3* (**F**) compared with the control and sham groups. There were no other significant differences in the mRNA expression of *Dnmt1* (**A**), *Dnmt3b* (C), and *Tet 2* (**E**). The medians are depicted using the horizontal lines inside the boxes. The 25th and 75th percentiles are shown as the bottoms and tops of the boxes, respectively. The minimum and maximum values are presented as the small horizontal lines below and above the boxes. *n* = 6 per group. Data were analyzed using one-way analysis of variance, followed by the Tukey’s *post hoc* test. *, *p* < 0.05; **, *p* < 0.01

The mRNA expression of *Dnmt 3a* in the OA group significantly increased compared with that in the control and sham groups (Fig. 5B).

#### Ten–eleven translocation family

The mRNA expression of *Tet 1* (Fig. 5D) and *Tet 3* (Fig. 5F) in the OA group was significantly higher than that in the control and sham groups.

### Global DNA methylation in the peripheral blood mononuclear cells and synovial tissue

**Fig. 6:**
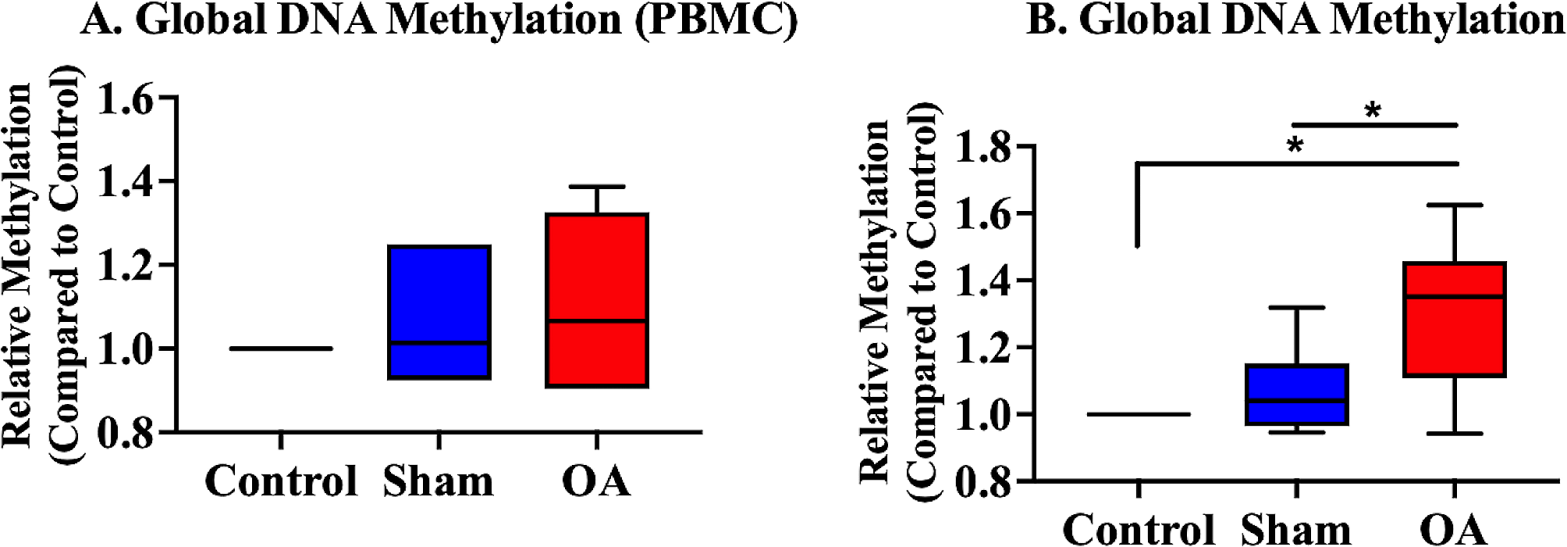
Global DNA methylation in the peripheral blood mononuclear cells and synovial tissues. There was no significant difference in global DNA methylation in the peripheral blood mononuclear cells among the control, sham, and OA groups. However, the OA group had a significantly higher global DNA methylation in the synovial tissue than in the control and sham groups. The medians are depicted using the horizontal lines inside the boxes. The 25th and 75th percentiles are shown as the bottoms and tops of the boxes, respectively. The minimum and maximum values are presented as the small horizontal lines below and above the boxes. *n* = 6 per group. Data were analyzed using one-way analysis of variance, followed by the Tukey’s *post hoc* test. *, *p* < 0.05. PBMC, peripheral blood mononuclear cell

Global methylation of the whole genomic DNA, including that from the CpG sites from promoter, coding, and noncoding sequences, was measured. The OA group had a significantly higher global DNA methylation in the synovial tissue than in the control and sham groups (Fig. 6B).

## Discussion

Injection of 2 mg of MIA into the hip joint resulted in the marked JSN, subchondral bone cyst formation, subchondral sclerosis, and femoral head deformity. Hence, mild to moderate OA based on the KL classification was observed. This study first investigated arthritic changes in rat hip joints using µCT scan images. Our previous studies using X-ray images reported that 2 mg of MIA administered in the hip joint can cause possible JSN and cystic changes [26, 29]. However, it was challenging to evaluate arthritic changes and subchondral bone sclerosis in the acetabulum. Therefore, this study used µCT scan to evaluate the hip joint. Yoh et al. performed radiographic assessment on rats at 4 weeks after the administration of 2 mg of MIA into the hip joints, and results showed the development of KL2-3 OA [33]. Another study investigated arthritic changes in MIA-induced shoulder OA on µCT scan. In total, 0.5 mg and 2.0 mg of MIA were injected into the shoulder joint, and µCT scan was performed from 1 to 12 weeks after induction. At 4 weeks after induction, 83% and 100% of rates who received 0.5 and 2.0 of MIA developed arthritis, and 67% and 83% presented with osteophyte development [38]. The study result supports these findings.

A previous study revealed that intra-articular MIA injection to the hip joints of rats induced diverse OA changes based on the dose, similar to previous studies on knee joints [33, 39]. Low-dose MIA caused fewer radiographic and histopathological OA changes than high-dose MIA. The low-dose groups experienced OA progression over time, and > 2 mg of MIA caused end-stage OA 8 weeks after injection. Therefore, osteoarthritic changes based on each concentration without any ceiling effect can be sufficiently assessed 4 weeks after MIA administration. In a previous study, the OARSI score at 4 weeks after the administration of MIA at a dose of 2 mg was 19.6 [33]. Miyamoto et al. examined cartilage degeneration using the Mankin score in MIA-induced hip OA. Results showed that severe cartilage degeneration occurred 4 weeks after induction [25, 40], and our results are consistent with these findings.

The OA group had a significantly lower pain threshold than the control and sham groups from 7 to 28 days after induction. In addition, none of the rats in the OA group were painless, and the pain could not be associated with other pathological conditions such as Charcot arthropathy. There was a significant difference between the control and sham groups on day 7 after MIA administration. Thereafter, this finding disappeared over time. Thus, it is reasonable to consider this phenomenon as a change in pain threshold due to surgical invasion. Several reports have revealed that the injection of MIA into the hip joint induces pain-related behavior in the affected limb. In 2016, the study revealed that the injection of 2 mg of MIA into the hip joint induced gait disturbance based on changes in various parameters such as standing time, swing speed, print area, and maximum intensity based on a gait analysis [41]. This study showed that 2 mg of MIA administered to the hip joint elicited hypersensitivity in the affected limb. More data on MIA-induced knee OA than MIA-induced hip OA have been collected. However, changes in pain-related behavior in MIA-induced knee OA are consistent with the abovementioned results in hip OA [42, 43]. The details of hypersensitivity observed in this study were also consistent with those of previous reports, and we believe that the reproducibility and validity of the model were adequate.

In the current study, the mRNA expression of *Dnmt 3a, Tet 1*, and *Tet 3* was significantly upregulated in the synovial tissue of the OA group. Global DNA methylation in the synovial tissue of the OA group was significantly higher than that of the control and sham groups. In contrast, only the mRNA expression of *Dnmt 1* and *Tet 1* in the PBMC tissues of the OA group increased compared with that in the sham group. In addition, there was no significant difference in global DNA methylation. No previous studies have investigated changes in DNA methylation patterns across tissue types in hip OA. Based on other reports, changes in DNA methylation patterns in local tissues that reflect the pathological condition are more apparent than changes in DNA methylation patterns in the peripheral blood [44, 45]. As with other diseases, alterations in the DNA methylation machinery in the local synovial and cartilage tissues may provide more informative findings on osteoarthritis than those in the peripheral blood.

Most genetic variations related to OA pathology occur in the noncoding regions of the genome. Thus, increasing studies have focused on epigenetics, particularly DNA methylation, which regulates gene expression without changing the gene sequence [22]. Accumulating scientific evidence has revealed significant changes in the epigenetic regulatory machinery in OA cartilage [46]. Articular cartilages from patients with OA and mice with destabilized medial meniscus showed aberrant elevations of DNMT 1 and DNMT 3a, resulting in consequential peroxisome proliferator-activated receptor-gamma (PPARγ) promoter hypermethylation and substantial PPARγ suppressions. Further, 5-Aza-2’-deoxycytidine, a known inhibitor of both DNMT 1 and DNMT 3a, reduced excessive methylation of the PPARγ promoter region in mice with OA, thereby suppressing the production of excessive inflammatory cytokines and, ultimately, cartilage damage [47]. In another study, DNMT 3a was found to be upregulated in patients with OA compared with controls. Furthermore, the knockdown of DNMT 3a reduced the catabolic effect of interleukin 1β (IL1β) on the extracellular matrix (ECM) [48]. By contrast, DNMT 3b has protective effects against OA changes in the cartilage. Shen et al. revealed that mice without DNMT 3b were more susceptible to spontaneous OA, and the increased expression of *Dnmt 3b* had protective effects against surgically induced OA in mice [49]. Interestingly, recent evidence has shown that DNMT 3b is involved in the pathogenesis of secondary OA caused by femoroacetabular impingement (FAI). In this study, the *DNMT 3b* mRNA expression was evaluated in cartilage samples collected from patients with early FAI and those with late FAI-OA. Further, the mRNA expression of *DNMT 3b* decreased as OA progressed from the normal cartilage to the early FAI-OA and between early FAI-OA and late FAI-OA [50]. Thus far, all reports on Dnmts have used cartilage samples, and only a few studies have analyzed the profiles of Dnmts in synovial tissue. However, the mRNA expression of Dnmt 3a was also upregulated in the OA group in our study. This result is consistent with the results of these studies using cartilage samples. Our result can be reasonable considering that OA pathology comprises the cartilage, synovium, and subchondral bone, which crosstalk with each other.

The DNA methylation machinery also includes the demethylation process by TET enzymes. Among the three bioactive TETs, TET1 is one of the major enzymes responsible for DNA demethylation in the chondrocytes and 5hmC generation in OA chondrocytes. Previous research has revealed that 5hmC accumulates in the OA-associated genes in the human osteoarthritic cartilage [51]. Genetic loss of TET1 inhibited OA in a surgically induced knee OA model in which the medial meniscus was destabilized to initiate OA pathology. They also analyzed human cartilage samples and revealed that TET1 was involved in cartilage homeostasis and the balance of anabolic versus catabolic factors in the ECM. The inhibition of TET1 modulated inflammation in end-stage human OA chondrocytes, which mirrored mouse studies [52]. Another study reported that twist basic helix-loop-helix transcription factor 1, a transcription factor overexpressed in OA, upregulated TET1 expression and 5hmC in chondrocytes and escalated matrix metalloproteinase-3 expression [53]. According to few reports analyzing noncartilage tissues, the expression of TET3 and 5hmC were upregulated in the synovial tissue from patients with RA. TNFα enhanced the TET3 and 5hmC levels in cultured fibroblast-like synoviocytes and the stimulated fibroblast-like synoviocyte amplified transcription of cell migration-related factors such as C-X-C motif chemokine ligand 8 and C-C motif chemokine ligand 2 in a TET 3-dependent manner, leading to high cell migration [54]. Our findings showed that the mRNA expression of *Tet 1* and *Tet 3* significantly increased in the synovial tissue of the OA group. This is consistent with the results of previous studies.

This study differed from others as it investigated the synovial tissue, an essential pathological component of OA, not the cartilage. To the best of our knowledge, to date, this is the only study that has investigated alterations in the DNA methylation machinery in the synovial tissue of a hip OA model. Furthermore, as mentioned above, epigenetics, including DNA methylation, is influenced by acquired factors such as diet and exercise. Further, previous studies using human specimens cannot completely eliminate the differences in each background. The use of the rat OA model in this study uniformed the background of the samples and provided a more detailed understanding of the effects of OA on the DNA methylation machinery. Notably, this study showed that the changes in global methylation and the mRNA expression of the DNA methylation-related genes were explicit in the synovial tissue. The application of epigenetic regulators such as DNMTs and TETs as pharmacological candidates for disease-modifying OA agents is interesting as it can simultaneously modify multiple regulated genes. However, a major challenge in directly modulating epigenetic regulators is to ensure specificity and prevent adverse effects. Drugs that target epigenetic regulators have been used primarily as cancer drugs [55], and their efficacy is widely recognized. However, the fact that these regulators can also affect unintended genes is a challenge. Therefore, future studies should focus on providing a better understanding on the exact target of epigenetic enzymes such as DNMT and TET in OA and their tissue-specific differences.

The current study has several limitations. First, changes in the DNA methylation machinery of the cartilage, which is significantly involved in OA pathology, were not investigated. MIA chemically induces cartilage degeneration by inhibiting the glycolytic pathway of chondrocytes. Thus, synovial tissues that were not directly affected by MIA administration were analyzed. Nevertheless, cartilage samples taken from surgically induced knee OA models will be analyzed. Second, the samples used were collected from rats only. Although acquiring uniform data from humans is challenging, further research with human tissues is required to develop these findings. Third, the DNA methylation and transcriptional status of individual genes that contribute directly to OA should be examined in future studies. As mentioned above, there are no reports on the methylation of individual genes in the synovial tissue of patients with hip OA. We are currently exploring methylation patterns in the synovial tissue from both humans and rats with OA by isolating specific cell types using flow cytometry. Fourth, the validation of the PCR assay was inadequate. Particularly, the RNA integrity number was good, and the assays were evaluated in the same tissue. However, the validation of the assay specificity, such as primer validation, and assay performance was insufficient. Hence, future studies should be conducted to address these issues by comparing human and rat samples.

## Conclusion

The intra-articular administration of 2 mg of MIA-induced radiological and histopathological hip joint OA and decreased pain threshold. The DNA methylation machinery was altered in hip OA. Specifically, the mRNA expression of *Dnmt 3a*, *Tet 1*, and *Tet 3* was significantly upregulated in the synovial tissue of the OA group. The global DNA methylation in the synovial tissue of the OA group was significantly high. A better understanding of alterations in the DNA methylation machinery in the synovial tissue of patients with OA can lead to the discovery of novel gene targets and treatment strategies against OA that are more tissue-specific and have fewer side effects.

## Data Availability

No datasets were generated or analysed during the current study.

## Abbreviations

JSN: Joint space narrowing
KL: Kellgren and Lawrence
OARSI: Osteoarthritis Research Society International
DNMT: DNA methyltransferase
ECM: Extracellular matrix
FAI: uc Femoroacetabular Impingement
MIA: Monoiodoacetate

## References

[1] GBD 2021 Osteoarthritis Collaborators (2023). Global, regional, and national burden of osteoarthritis, 1990–2020 and projections to 2050: a systematic analysis for the global burden of Disease Study 2021. Lancet Rheumatol.

[2] Vina ER, Kwoh CK (2018). Epidemiology of osteoarthritis: literature update. Curr Opin Rheumatol.

[3] Ferre IM, Roof MA, Anoushiravani AA, Wasterlain AS, Lajam CM (2019). Understanding the observed sex discrepancy in the prevalence of Osteoarthritis. JBJS Rev.

[4] Callahan LF, Cleveland RJ, Allen KD, Golightly Y, Racial/Ethnic (2021). Socioeconomic, and Geographic Disparities in the epidemiology of knee and hip osteoarthritis. Rheum Dis Clin North Am.

[5] Ding C, Yimiti D, Sanada Y, Matsubara Y, Nakasa T, Matsubara K et al. High-fat diet-induced obesity accelerates the progression of spontaneous osteoarthritis in senescence-accelerated mouse prone 8 (SAMP8). Mod Rheumatol. 2023. 10.1093/mr/road069 37522619

[6] Xu C, Wang S, Ti W, Yang J, Yasen Y, Memetsidiq M (2022). Role of dietary patterns and factors in determining the risk of knee osteoarthritis: a meta-analysis. Mod Rheumatol.

[7] Poulsen E, Goncalves GH, Bricca A, Roos EM, Thorlund JB, Juhl CB (2019). Knee osteoarthritis risk is increased 4–6 fold after knee injury - a systematic review and meta-analysis. Br J Sports Med.

[8] Canetti EFD, Schram B, Orr RM, Knapik J, Pope R (2020). Risk factors for development of lower limb osteoarthritis in physically demanding occupations: a systematic review and meta-analysis. Appl Ergon.

[9] Valdes AM, Spector TD (2011). Genetic epidemiology of hip and knee osteoarthritis. Nat Rev Rheumatol.

[10] Waheed A, Rai MF. Osteoarthriris year in review 2023: genetics, genomics, and epigenetics. Osteoarthr Cartil. 2023. 10.1016/j.joca.2023.11.006 37979669

[11] Henikoff S, Smith MM (2015). Histone variants and epigenetics. Cold Spring Harb Perspect Biol.

[12] Richardson BC, Patel DR (2014). Epigenetics in 2013. DNA methylation and miRNA: key roles in systemic autoimmunity. Nat Rev Rheumatol.

[13] Voisin S, Eynon N, Yan X, Bishop DJ (2015). Exercise training and DNA methylation in humans. Acta Physiol (Oxf).

[14] Vineis P, Chatziioannou A, Cunliffe VT, Flanagan JM, Hanson M, Kirsch-Volders M (2017). Epigenetic memory in response to environmental stressors. FASEB J.

[15] Gao L, Emperle M, Guo Y, Grimm SA, Ren W, Adam S (2020). Comprehensive structure-function characterization of DNMT3B and DNMT3A reveals distinctive de novo DNA methylation mechanisms. Nat Commun.

[16] Niederberger E, Resch E, Parnham MJ, Geisslinger G (2017). Drugging the pain epigenome. Nat Rev Neurol.

[17] Singh P, Lessard SG, Mukherjee P, Carballo CB, Rodeo SA, Otero M (2019). The progression of post-traumatic osteoarthritis in the murine DMM model is marked by distinctive epigenomic patterns associated with transcriptomic changes in articular cartilage. Osteoarthr Cartil.

[18] Milagro FI, Campión J, García-Díaz DF, Goyenechea E, Paternain L, Martínez JA (2009). High fat diet-induced obesity modifies the methylation pattern of leptin promoter in rats. J Physiol Biochem.

[19] Miranda-Duarte A, Borgonio-Cuadra VM, González-Huerta NC, Rojas-Toledo EX, Ahumada-Pérez JF, Sosa-Arellano M (2020). DNA methyltransferase genes polymorphisms are associated with primary knee osteoarthritis: a matched case-control study. Rheumatol Int.

[20] Aubourg G, Rice SJ, Bruce-Wootton P, Loughlin J (2022). Genetics of osteoarthritis. Osteoarthr Cartil.

[21] Grandi FC, Bhutani N (2020). Epigenetic therapies for Osteoarthritis. Trends Pharmacol Sci.

[22] Rice SJ, Beier F, Young DA, Loughlin J (2020). Interplay between genetics and epigenetics in osteoarthritis. Nat Rev Rheumatol.

[23] de Andres MC, Imagawa K, Hashimoto K, Gonzalez A, Roach HI, Goldring MB (2013). Loss of methylation in CpG sites in the NF-kappaB enhancer elements of inducible nitric oxide synthase is responsible for gene induction in human articular chondrocytes. Arthritis Rheum.

[24] Bradley EW, Carpio LR, McGee-Lawrence ME, Castillejo Becerra C, Amanatullah DF, Ta LE (2016). Phlpp1 facilitates post-traumatic osteoarthritis and is induced by inflammation and promoter demethylation in human osteoarthritis. Osteoarthr Cartil.

[25] Miyamoto S, Nakamura J, Ohtori S, Orita S, Omae T, Nakajima T (2016). Intra-articular injection of mono-iodoacetate induces osteoarthritis of the hip in rats. BMC Musculoskelet Disord.

[26] Kawarai Y, Orita S, Nakamura J, Miyamoto S, Suzuki M, Inage K (2018). Changes in proinflammatory cytokines, neuropeptides, and microglia in an animal model of monosodium iodoacetate-induced hip osteoarthritis. J Orthop Res.

[27] Kanno K, Suzuki-Narita M, Kawarai Y, Hagiwara S, Yoh S, Nakamura J et al. Analgesic effects and arthritic changes following tramadol administration in a rat hip osteoarthritis model. J Orthop Res. 2021. 10.1002/jor.25208 34783063

[28] Charan J, Kantharia ND (2013). How to calculate sample size in animal studies?. J Pharmacol Pharmacother.

[29] Kawarai Y, Orita S, Nakamura J, Miyamoto S, Suzuki M, Inage K (2020). Analgesic effect of Duloxetine on an animal model of Monosodium Iodoacetate-Induced Hip Osteoarthritis. J Orthop Res.

[30] Omae T, Nakamura J, Ohtori S, Orita S, Yamauchi K, Miyamoto S (2015). A novel rat model of hip pain by intra-articular injection of nerve growth factor-characteristics of sensory innervation and inflammatory arthritis. Mod Rheumatol.

[31] Zhang J, Li J, Qu X, Liu Y, Harada A, Hua Y, Yoshida N, Ishida M, Tabata A, Sun L, Liu L, Miyagawa S (2023). Development of a thick and functional human adipose-derived stem cell tissue sheet for myocardial infarction repair in rat hearts. Stem Cell Res Ther.

[32] Kellgren JH, Lawrence JS (1957). Radiological assessment of osteo-arthrosis. Ann Rheum Dis.

[33] Yoh S, Kawarai Y, Hagiwara S, Orita S, Nakamura J, Miyamoto S (2022). Intra-articular injection of monoiodoacetate induces diverse hip osteoarthritis in rats, depending on its dose. BMC Musculoskelet Disord.

[34] Pritzker KP, Gay S, Jimenez SA, Ostergaard K, Pelletier JP, Revell PA (2006). Osteoarthritis cartilage histopathology: grading and staging. Osteoarthr Cartil.

[35] Millecamps M, Tajerian M, Sage EH, Stone LS (2011). Behavioral signs of chronic back pain in the SPARC-null mouse. Spine (Phila Pa 1976).

[36] Chaplan SR, Bach FW, Pogrel JW, Chung JM, Yaksh TL (1994). Quantitative assessment of tactile allodynia in the rat paw. J Neurosci Methods.

[37] Bustin SA, Benes V, Garson JA, Hellemans J, Huggett J, Kubista M (2009). The MIQE guidelines: minimum information for publication of quantitative real-time PCR experiments. Clin Chem.

[38] Ise S, Ochiai N, Hashimoto E, Hirosawa N, Kajiwara D, Shimada Y (2023). Evaluation of articular changes using a rat mono-iodoacetate-induced shoulder arthritis model by histology and radiology. J Orthop Res.

[39] Longo UG, Loppini M, Fumo C, Rizzello G, Khan WS, Maffulli N, Denaro V (2012). Osteoarthritis: new insights in animal models. Open Orthop J.

[40] Mankin HJ, Dorfman H, Lippiello L, Zarins A (1971). Biochemical and metabolic abnormalities in articular cartilage from osteo-arthritic human hips. II. Correlation of morphology with biochemical and metabolic data. J Bone Joint Surg Am.

[41] Miyamoto S, Nakamura J, Ohtori S, Orita S, Nakajima T, Omae T (2017). Pain-related behavior and the characteristics of dorsal-root ganglia in a rat model of hip osteoarthritis induced by mono-iodoacetate. J Orthop Res.

[42] Arai T, Suzuki-Narita M, Takeuchi J, Tajiri I, Inage K, Kawarai Y (2022). Analgesic effects and arthritic changes following intra-articular injection of diclofenac etalhyaluronate in a rat knee osteoarthritis model. BMC Musculoskelet Disord.

[43] Orita S, Ishikawa T, Miyagi M, Ochiai N, Inoue G, Eguchi Y (2011). Pain-related sensory innervation in monoiodoacetate-induced osteoarthritis in rat knees that gradually develops neuronal injury in addition to inflammatory pain. BMC Musculoskelet Disord.

[44] Lin PI, Shu H, Mersha TB (2020). Comparing DNA methylation profiles across different tissues associated with the diagnosis of pediatric asthma. Sci Rep.

[45] Nelson JS, Kwok C, Braganca NE, Lopez DL, Espina Rey AP, Robinson M, Ebert SN (2022). Comparison of DNA methylation patterns across tissue types in infants with tetralogy of Fallot. Birth Defects Res.

[46] Miranda-Duarte A (2018). DNA methylation in Osteoarthritis: current status and therapeutic implications. Open Rheumatol J.

[47] Zhu X, Chen F, Lu K, Wei A, Jiang Q, Cao W (2019). PPARgamma preservation via promoter demethylation alleviates osteoarthritis in mice. Ann Rheum Dis.

[48] Ma F, Li G, Yu Y, Xu J, Wu X (2019). MiR-33b-3p promotes chondrocyte proliferation and inhibits chondrocyte apoptosis and cartilage ECM degradation by targeting DNMT3A in osteoarthritis. Biochem Biophys Res Commun.

[49] Shen J, Wang C, Li D, Xu T, Myers J, Ashton JM et al. DNA methyltransferase 3b regulates articular cartilage homeostasis by altering metabolism. JCI Insight. 2017;2(12). 10.1172/jci.insight.93612 PMC5470890 28614801

[50] Kamenaga T, Shen J, Wu M, Brophy RH, Clohisy JC, O’Keefe RJ (2023). Epigenetic dysregulation of articular cartilage during progression of hip femoroacetabular impingement disease. J Orthop Res.

[51] Taylor SE, Smeriglio P, Dhulipala L, Rath M, Bhutani N (2014). A global increase in 5-hydroxymethylcytosine levels marks osteoarthritic chondrocytes. Arthritis Rheumatol.

[52] Smeriglio P, Grandi FC, Davala S, Masarapu V, Indelli PF, Goodman SB et al. Inhibition of TET1 prevents the development of osteoarthritis and reveals the 5hmC landscape that orchestrates pathogenesis. Sci Transl Med. 2020;12(539). 10.1126/scitranslmed.aax2332 32295898

[53] Hasei J, Teramura T, Takehara T, Onodera Y, Horii T, Olmer M (2017). TWIST1 induces MMP3 expression through up-regulating DNA hydroxymethylation and promotes catabolic responses in human chondrocytes. Sci Rep.

[54] Kawabe A, Yamagata K, Kato S, Nakano K, Sakata K, Tsukada YI (2022). Role of DNA dioxygenase ten-Eleven translocation 3 (TET3) in rheumatoid arthritis progression. Arthritis Res Ther.

[55] Mohammad HP, Barbash O, Creasy CL (2019). Targeting epigenetic modifications in cancer therapy: erasing the roadmap to cancer. Nat Med.

